# Ocular Equivocation: The Rivalry Between Wheatstone and Brewster

**DOI:** 10.3390/vision3020026

**Published:** 2019-06-06

**Authors:** Nicholas J. Wade

**Affiliations:** Department of Psychology, University of Dundee, Nethergate, Dundee DD1 4HN, UK; n.j.wade@dundee.ac.uk

**Keywords:** Wheatstone, Brewster, Chimenti, binocular rivalry

## Abstract

Ocular equivocation was the term given by Brewster in 1844 to binocular contour rivalry seen with Wheatstone’s stereoscope. The rivalries between Wheatstone and Brewster were personal as well as perceptual. In the 1830s, both Wheatstone and Brewster came to stereoscopic vision armed with their individual histories of research on vision. Brewster was an authority on physical optics and had devised the kaleidoscope; Wheatstone extended his research on audition to render acoustic patterns visible with his kaleidophone or phonic kaleidoscope. Both had written on subjective visual phenomena, a topic upon which they first clashed at the inaugural meeting of the British Association for the Advancement of Science in 1832 (the year Wheatstone made the first stereoscopes). Wheatstone published his account of the mirror stereoscope in 1838; Brewster’s initial reception of it was glowing but he later questioned Wheatstone’s priority. They both described investigations of binocular contour rivalry but their interpretations diverged. As was the case for stereoscopic vision, Wheatstone argued for central processing whereas Brewster’s analysis was peripheral and based on visible direction. Brewster’s lenticular stereoscope and binocular camera were described in 1849. They later clashed over Brewster’s claim that the Chimenti drawings were made for a 16th-century stereoscope. The rivalry between Wheatstone and Brewster is illustrated with anaglyphs that can be viewed with red/cyan glasses and in Universal Freeview format; they include rivalling ‘perceptual portraits’ as well as examples of the stimuli used to study ocular equivocation.

## 1. Introduction

Charles Wheatstone (1802–1875) and David Brewster (1781–1868) were pioneers of research on binocular vision but they did not see eye-to-eye on interpretations of stereoscopic vision or on the history of its study. It is fitting, therefore, that they both examined binocular rivalry with the aid of Wheatstone’s mirror stereoscope. When different patterns are presented to corresponding regions of each eye, they compete with one another for visibility and the ensuing percepts vary over time. Descriptions of this phenomenon have a long history [1], and it is now called binocular rivalry. Brewster [2] referred to it thus: “The *ocular equivocation*, as it may be called, which is produced by the capricious disappearance and reappearance of images formed on nearly corresponding points of each eye, is placed beyond a doubt by Mr Wheatstone’s own experiments” (p. 359). That is, binocular contour rivalry could be studied more systematically with the aid of a stereoscope, the device displayed to the public by Wheatstone in 1838 [3,4].

Although Wheatstone’s announcement of the mirror stereoscope and his analysis of stereoscopic vision were made in 1838 [3], both mirror and prism stereoscopes were constructed for him as early as 1832 [4,5,6]. It was Wheatstone’s colleague at King’s College, London, the physiologist Herbert Mayo, who provided the first published account of the stereoscope [7]. Mirror and prism stereoscopes were made for Wheatstone by Murray and Heath, optical instrument makers in London. Brewster announced his more popular (lenticular) stereoscope with paired half-lenses in 1849 [8]; the first instrument was made in Dundee by George Lowdon [9]. The optical manipulation of disparities was also achieved in the 1850s with Wheatstone’s pseudoscope [10], which reversed them, and with Helmholtz’s telestereoscope [11], which exaggerated them. Using red/blue glasses to view similarly printed patterns was introduced in 1853 by Rollmann [12] although it was not widely adopted until later in the century. Rollmann referred to it as a “Colour stereoscope, comprised of a coloured double drawing and two coloured glasses” [12] (p. 187). In general, all the stereoscopes were enlisted to view stereoscopic images, particularly paired photographs, rather than to examine binocular rivalry.

In 1838, Wheatstone unveiled his stereoscope first at a meeting of the Royal Society in London and later at the British Association for the Advancement of Science held in Newcastle. Brewster’s initial reception of the stereoscope when he saw it at the Newcastle meeting was very positive: “it is one of the most valuable optical papers which has been presented to the Section” [13]. By 1844, his views had changed and he stated that “these extraordinary results are obviously subversive of the established laws of vision, but especially of the law of visible direction; and if they are true, they must arise from a sudden change in the properties of the humours, or in the functions of the retina” [2] (p. 357). This conclusion applied both to stereoscopic vision and binocular rivalry. It displayed clearly Brewster’s peripheral (retinal) interpretation of binocular vision in contrast to Wheatstone’s conclusion that they were based on central processes [4]. Wheatstone referred to the distinction as one between physical and mental philosophy [14,15,16,17]. Fourteen years after his first memoir, Wheatstone [10] published his second in which he described and illustrated an adjustable mirror stereoscope, a prism stereoscope, and a pseudoscope for reversing disparities. It was in this article that he drew an explicit distinction between mental and physical philosophy; that is, between psychology and physics, and he placed binocular vision in the province of psychology.

## 2. Binocular Rivalry

**Figure 1:**
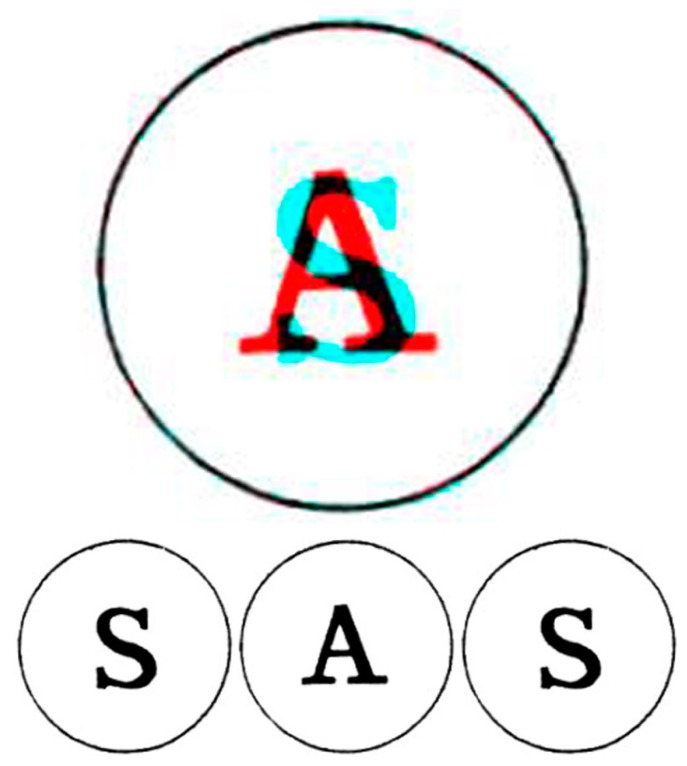
Rivalry between the letters S and A (from [3]). Upper: an anaglyph in which the rivalry can be seen with red/cyan glasses. Lower: left eye, right eye and left eye members of the binocular pairs presented in Universal Freeview format. There are two ways to superimpose the two rivalling letters: to cross-fuse the right pair, or to uncross-fuse the left pair.

Wheatstone examined rivalry between the letters A and S each surrounded by a similar circle (Figure 1) presented in the stereoscope and reported that “the common border will remain constant, while the letter within it will change alternately from that which would be perceived by the right eye alone to that which would be perceived by the left eye alone. At the moment of change the letter which has just been seen breaks into fragments, while the fragments of the letter which is about to appear mingle with them, and are immediately after replaced by the entire letter. It does not appear to be in the power of the will to determine the appearance of either of the letters, but the duration of the appearance seems to depend on causes which are under our control: thus if the two pictures be equally illuminated, the alternations appear in general of equal duration; but if one picture be more illuminated than the other, that which is less so will be perceived during a shorter time” [3] (p. 386).

In Figure 1 and in most subsequent figures, the rivalry pairs are presented in three ways. The upper image is an anaglyph combining the left and right images; the rivalry can be seen with the red filter of red/cyan glasses in front of the left eye and the cyan filter before the right eye or vice versa. Beneath the anaglyphs are three images presented in Universal Freeview format with those for the left eye flanking that for the right. The central and right images can be combined by ‘crossed convergence’ whereas the left and central images can be combined with ‘uncrossed viewing’.

**Figure 2:**
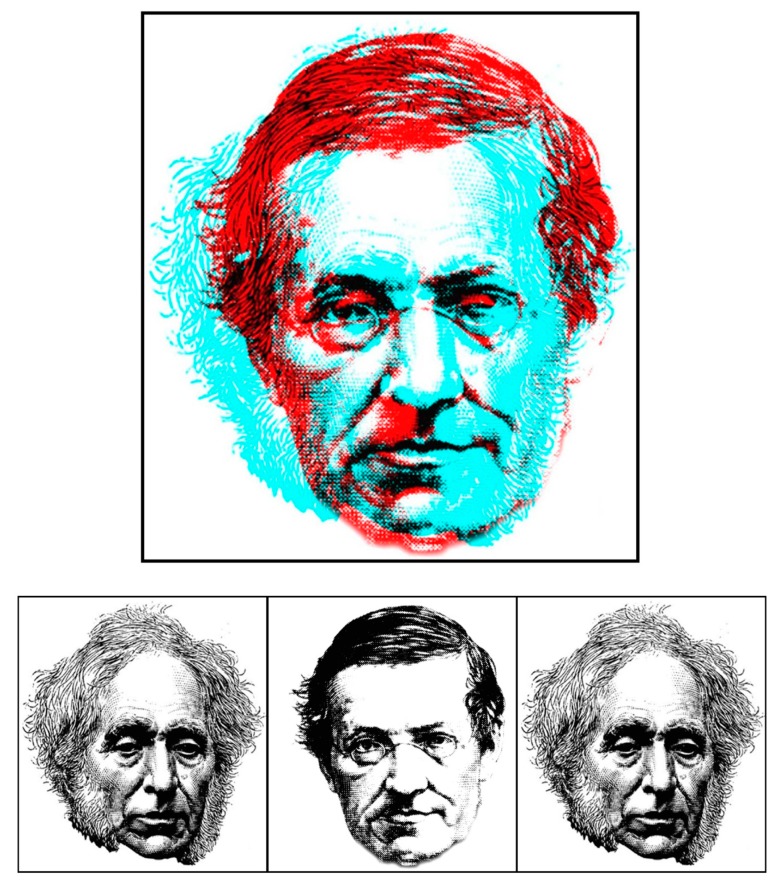
*Rivalling portraits of David Brewster and Charles Wheatstone* by Nicholas Wade.

Wheatstone and Brewster had competing interpretations of binocular rivalry as well as on stereoscopic depth perception, and their portraits engage in rivalry in Figure 2. Their descriptions of rivalry between the letters A and S were similar but another stimulus was the source of much disagreement between them. When a thick vertical line was presented to one eye and a thin vertical and a thick inclined one to the other (Figure 23 in [3]), Wheatstone reported that the two thick lines fused and were seen in depth but the thin vertical line was in the plane of the paper. His conclusion was that “This experiment affords another proof that there is no necessary physiological connection between the corresponding points of the two retinae—a doctrine which has been maintained by so many authors” (p. 384). Most of these authors were German and they responded vociferously after Wheatstone’s article was translated into German [18]. On the one hand, Wheatstone argued against the prevailing view of single vision advanced by Vieth [19] and Müller [20], and on the other it presented an empiricist interpretation of binocular vision. Particular opposition was directed to the suggestion that “similar pictures falling on corresponding points of the two retinae may appear double and in different places” [3] (p. 384) and Hering [21] referred to it as the ‘Wheatstone experiment’. The experiment was repeated by several German vision scientists whose observations differed from Wheatstone’s [15]. Brewster [2] also took issue with Wheatstone’s observations and wrote: “The phenomenon described by Mr. Wheatstone is an illusion, arising from actual disappearance of one or more parts, or even of the whole of one of the lines” [2] (p. 358). However, the disputes were generally about the depth seen in the display rather than the rivalry it induced.

**Figure 3:**
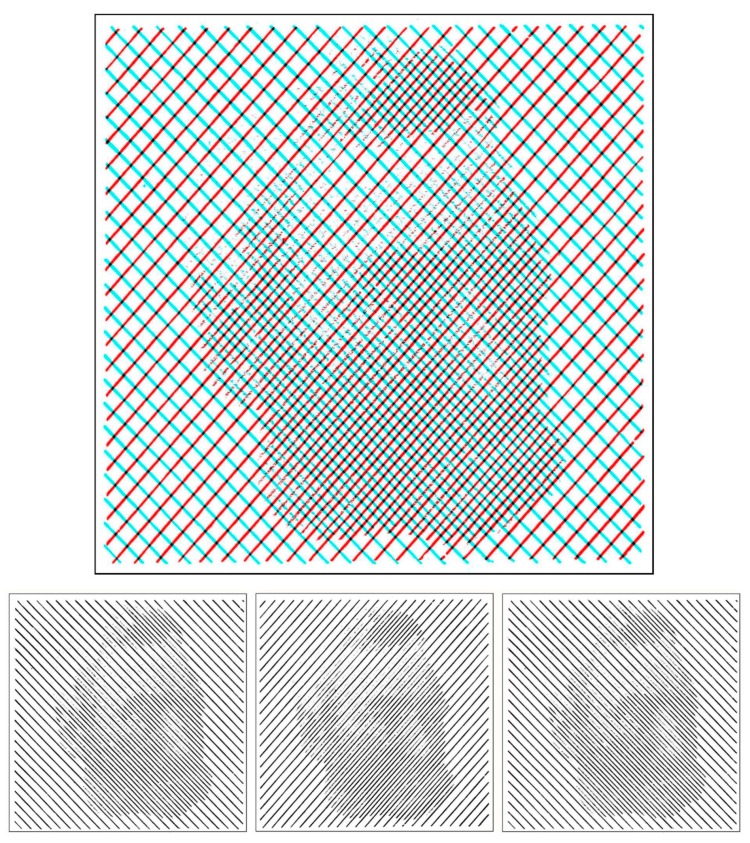
*Panum’s rivalry areas* by Nicholas Wade. A portrait of Panum is combined with the orthogonal gratings he illustrated in his book on vision with two eyes [22].

Panum [22] sought to reconcile stereoscopic phenomena with the Vieth–Müller circle by proposing the concept of fusional areas that now bear his name. Panum also examined binocular rivalry and introduced the stimulus that has been employed more than others in its study—orthogonal gratings (Figure 3). Not only did they produce strong rivalry but he also found it difficult to represent the changes that were experienced: Sometimes either one of the gratings were briefly visible, but changing mosaic-like composites of the two gratings were seen most of the time. Panum interpreted binocular contour rivalry in terms of physiological rather than psychological processes.

Orthogonal gratings became the standard stimulus for examining binocular rivalry thereafter. They were employed by Helmholtz [23] in order to support empiricist theories like that proposed by Wheatstone rather than the physiologically-based theories of Panum and Hering. Orthogonal gratings were also used by Breese [24] who introduced quantification into studies of binocular rivalry [1].

## 3. Personal Rivalry

Both Wheatstone and Brewster came to the study of binocular vision armed with their individual histories of research on vision. Brewster was an authority on physical optics and on polarisation in particular. His binocular optics were based on the concept of monocular visible direction and he raised it to a law: “We know nothing more than that the mind, residing, as it were, in every point on the retina, refers the impression made upon it at each point to a direction coinciding with the last portion of the ray which conveys the impression” [25] (p. 615). This ‘law’ was stated in his long article on optics for the *Edinburgh Encyclopædia* in 1830, before stereoscopic phenomena had been demonstrated. The article formed the basis of his book on optics [26] which ran to many editions. He invented the kaleidoscope in 1815 about which he wrote a book [27], and he published descriptions of a variety of visual phenomena, like afterimages [28], the colours and pattern distortions seen in finely ruled black-and-white gratings [29], and the reversed depth seen in hollow objects such as masks [30].

**Figure 4:**
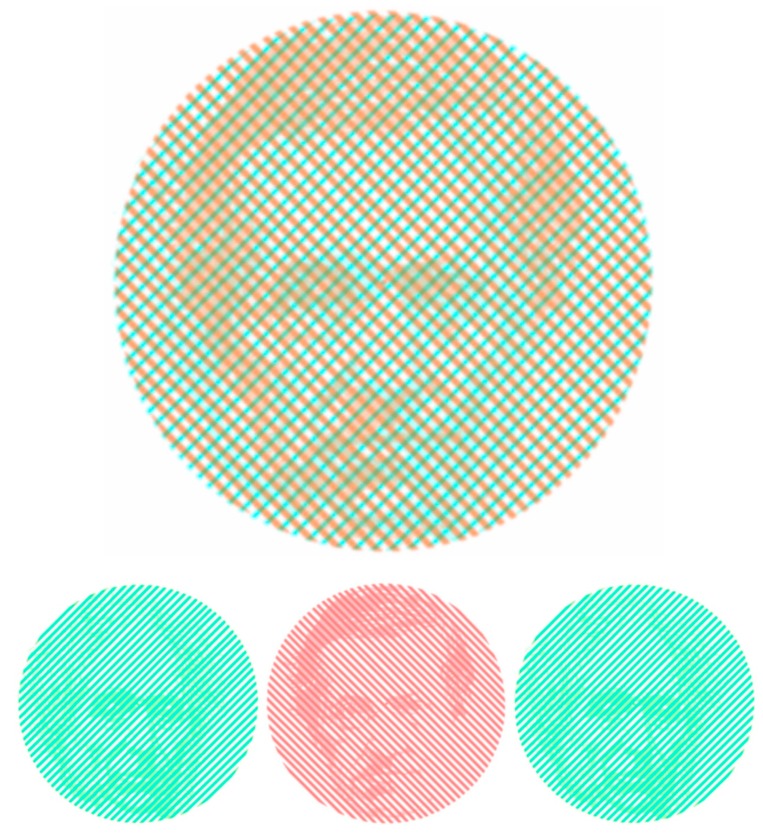
*Rivalling researchers* by Nicholas Wade. Portraits of Wheatstone and Brewster are embedded in orthogonal gratings.

Wheatstone came to vision from his work on acoustics for the family business of musical instrument manufacture [31]. This appealed to Wheatstone’s mechanical ingenuity, and among his inventions was the concertina. His first scientific paper was on acoustical figures [32], in which he also investigated binaural hearing. He extended his research on audition to render acoustic patterns visible with his kaleidophone or phonic kaleidoscope [33]. He provided a translated summary of Purkinje’s book on subjective visual phenomena and described a better method of rendering visible shadows of the retinal blood vessels [34]. Thus, both had written on subjective visual phenomena, a topic upon which they first clashed at the inaugural meeting of the British Association for the Advancement of Science in 1832 [4]. However, the rivalry between Wheatstone and Brewster remained theoretical until after the invention of the stereoscope when Brewster appreciated the implications of stereoscopic phenomena to his theory of vision. Thereafter, Brewster sought every opportunity to diminish the importance of Wheatstone’s invention. Their portraits are presented embedded in rivalling gratings in Figure 4.

**Figure 5:**
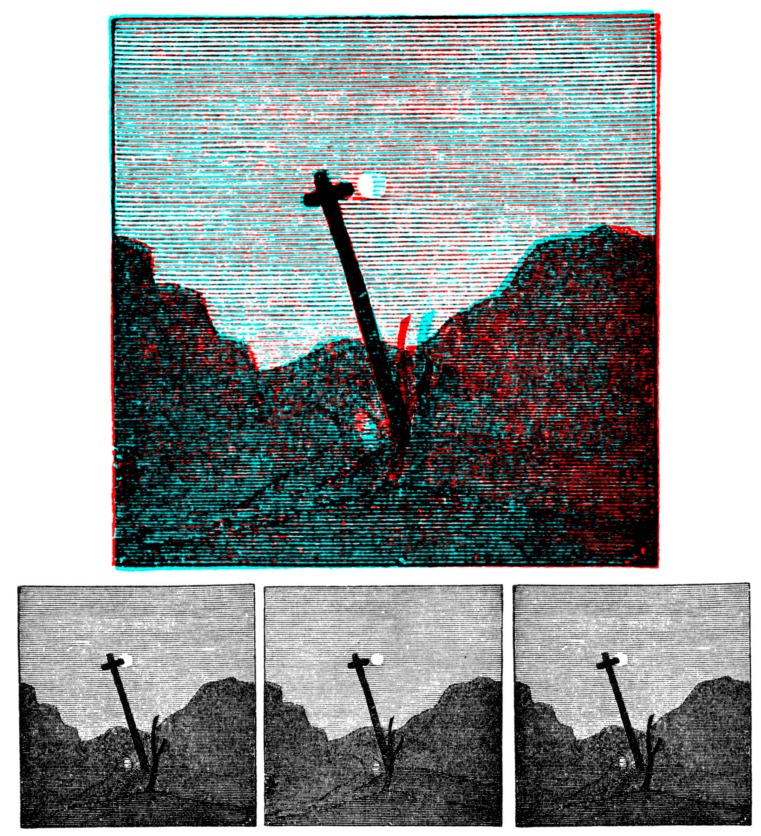
Elliot’s stereoscopic landscape. The lower left and centre images are from Brewster [35].

Brewster’s positive reception of Wheatstone’s mirror stereoscope turned to bitter acrimony with publication of Brewster’s [35] book *The stereoscope. Its history, theory, and construction*. He tried to wrest the invention of the stereoscope from Wheatstone, claiming that an ‘ocular stereoscope’ had been invented by James Elliot, an Edinburgh teacher of mathematics, in 1834. Brewster reproduced the stereopair attributed to Elliot to bolster his claim (Figure 5). The claim was repeated in an anonymous letter to *The Times* later in 1856. The correspondence between Wheatstone and Brewster is reprinted in Wade [4]; it did have the virtue of establishing that both mirror and prism stereoscopes were made for Wheatstone in 1832. While Elliot [36] retracted the claim made on his behalf, Brewster was not so repentant.

**Figure 6:**
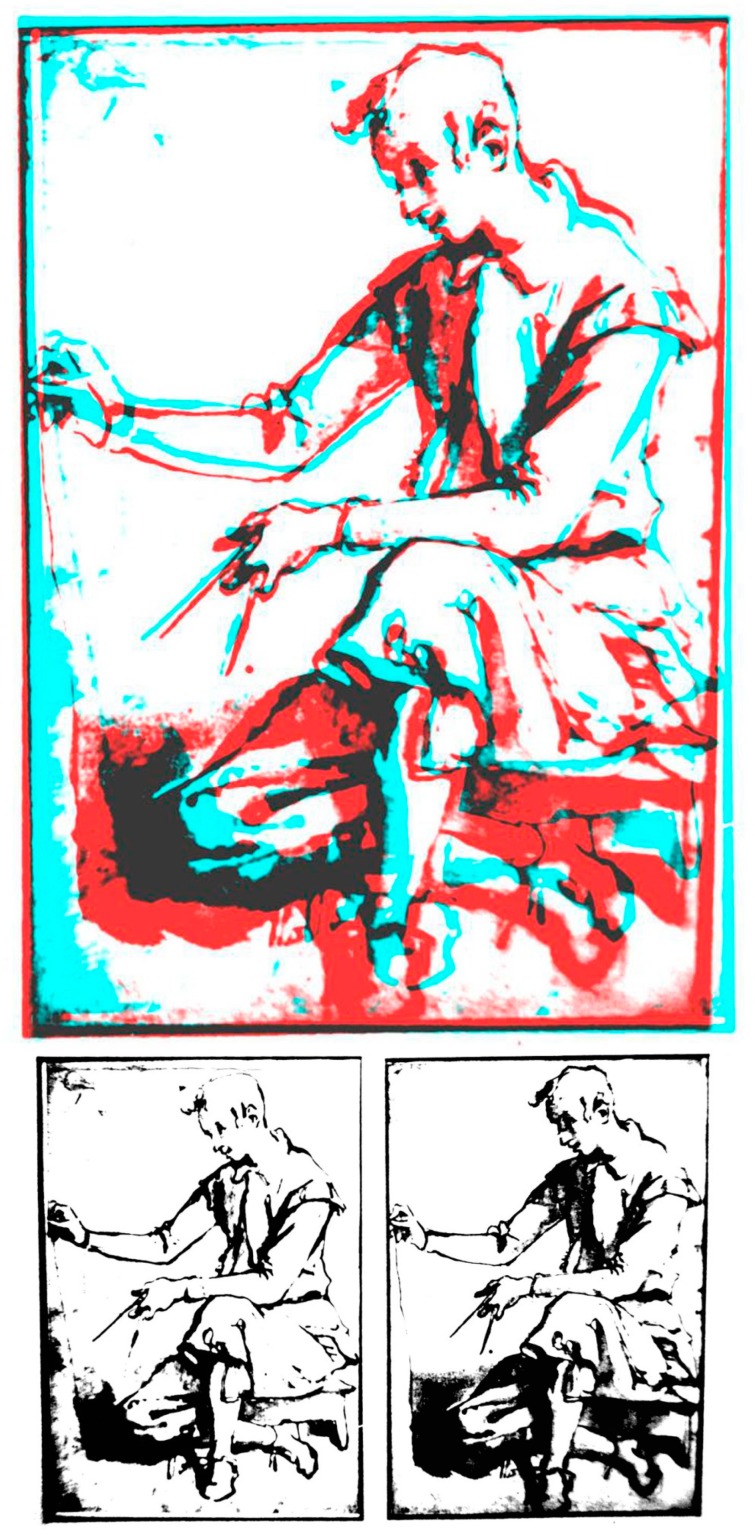
The Chimenti drawings derived from photographs taken by Arthur Gill. It is not clear whether there was a left and right image and so they are not presented in Universal Freeview format.

Not content with raising Elliot’s spurious claim to cast doubt on Wheatstone’s invention of the stereoscope, Brewster returned to the fray when he was informed of two sketches of a young man holding a compass and a plumb line (Figure 6) by Jacopo Chimenti (1551–1640), an artist from Empoli in Tuscany [37]. According to Gill [38], they were shown on separate sheets and there was no evidence that they had ever been mounted as a pair. Woodcuts from photographs were published by Reade [39] and are reproduced as anaglyphs in Brooks [40].

In 1859, the drawings, which were displayed side by side in the Musée Wicar (Lille), were seen by Alexander Crum Brown, then a medical student from Edinburgh. Crum Brown viewed the drawings with crossed eyes and described the depth he saw. He assumed Chimenti intended that the drawings should be viewed in this way. Crum Brown conveyed his observations on the drawings in a letter to James Forbes who passed it on to Brewster [37]. In his reply, Brewster indicated that he had requested a photograph of the Chimenti drawings. Brewster [41] published Crum Brown’s letter and added his own comments concluding: “This account of the two drawings is so distinct and evinces such knowledge of the subject, that we cannot for a moment doubt that they are binocular drawings intended by the artist to be united into relief either by the eye or by an instrument” [41] (p. 233). Brewster had not seen the drawings and the claim that they were stereoscopic rested on Crum Brown’s account. Despite this lack of evidence, Brewster made reference to Chimenti’s drawings in his entry on the “Stereoscope” for the 8th edition of the *Encyclopædia Britannica* [42].

Brewster’s chagrin must have been heightened on discovering that Wheatstone had obtained photographic copies of the drawings in June 1860; although he did not publish his comments on them, his London colleagues did cast doubt on the stereoscopic effects seen in the drawings [39,43]. Carpenter displayed Wheatstone’s photographic copies in a lecture delivered at the London Institution and he showed them to “some of the most eminent photographers” in London; none were convinced that they were stereoscopic. Artists who Carpenter enlisted to view the photographs suggested that one “is the work of the master and the other an inferior copy by a pupil” [43] (p. 11).

The ‘Chimenti controversy’ ensnared many of the leading visual scientists in Europe and even those across the Atlantic [37]. Indeed, it was an American, Edwin Emerson [44], who essentially resolved the controversy by measuring the dimensions of the two drawings in detail, and a colleague did so independently. He found that there was a melange of stereoscopic and pseudoscopic disparities between the drawings that did not indicate any systematic depth. Emerson’s conclusion has been confirmed by Brooks [40] who presented copies of the Chimenti drawings to naïve observers and found no consistent ratings of depth. The most likely interpretations of the paired pictures are that Chimenti either made two drawings of the boy in sequence or made a copy of the sketch. In both cases, disparities would have been introduced by the inaccuracies of drawing but they would not have been consistent. In *The Times* correspondence over the invention of the stereoscope, Wheatstone wrote: “I have hitherto avoided entangling myself in the meshes of controversy with so disputatious an antagonist as Sir David Brewster. I have always thought myself more usefully employed in investigating new facts, than in contending in respecting errors which time will inevitably correct” (reprinted in [4] p. 181). In the cases of both Elliot’s stereoscope and the Chimenti drawings, time has indeed found in Wheatstone’s favour. Perhaps the most fitting epitaph to this sorry saga was delivered by Reade: “The eye is a treacherous guide when fortified by a little previous theory” [39] (p. 29).

## 4. Conclusions

Wheatstone and Brewster had no disputes about the optics of projections to the two eyes. Brewster made several such diagrams from 1830 onwards, and particularly in *The stereoscope* [35]; Wheatstone made no such illustrations. Their theoretical disputes about binocular rivalry were concerned with the sites at which depth and rivalry took place. Wheatstone adopted the methods of the physical sciences to support empiricist theories of perception of the type proposed by Berkeley [45] and pursued by Helmholtz [23]. Brewster also employed the methods of physics but his theories were physically based, too. He had little sympathy for Berkeley’s approach to perception and often criticized it. With Brewster’s background in optics, he would have been the one expected to devise a stereoscope, and this was probably a factor in his hostility towards Wheatstone. Brewster’s views did not change with knowledge of stereoscopic phenomena, because for him the laws of binocular vision had to comply with those of monocular vision. Whereas Brewster was confounded by the phenomenal complexities of binocular rivalry, they were seen as no impediment to Wheatstone’s inferential theory.

**Figure 7:**
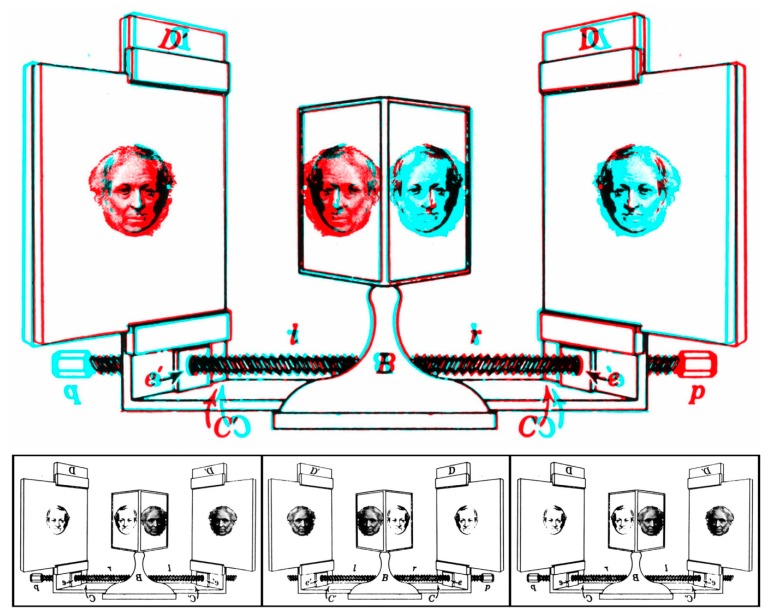
*Ocular equivocation* by Nicholas Wade. Rivalling portraits of Wheatstone and Brewster are shown mounted in Wheatstone’s stereoscope.

Wheatstone and Brewster are shown engaging in ocular equivocation in Figure 7.
